# Municipal biowaste treatment plants contribute to the contamination of the environment with residues of biodegradable plastics with putative higher persistence potential

**DOI:** 10.1038/s41598-022-12912-z

**Published:** 2022-05-30

**Authors:** Thomas Steiner, Yuanhu Zhang, Julia N. Möller, Seema Agarwal, Martin G. J. Löder, Andreas Greiner, Christian Laforsch, Ruth Freitag

**Affiliations:** 1 Process Biotechnology, University of Bayreuth, Universitätsstrasse 30, 95440 Bayreuth, Germany; 2 Macromolecular Chemistry II, University of Bayreuth, Bayreuth, Germany; 3 Animal Ecology I & BayCEER, University of Bayreuth, Bayreuth, Germany

**Keywords:** Ecology, Environmental sciences

## Abstract

Biodegradable plastics (BDP) are expected to mineralize easily, in particular under conditions of technical composting. However, the complexity of the sample matrix has largely prevented degradation studies under realistic conditions. Here composts and fertilizers from state-of-the-art municipal combined anaerobic/aerobic biowaste treatment plants were investigated for residues of BDP. We found BDP fragments > 1 mm in significant numbers in the final composts intended as fertilizer for agriculture and gardening. Compared to pristine compostable bags, the recovered BDP fragments showed differences in their material properties, which potentially renders them less prone to further biodegradation. BDP fragments < 1 mm were extracted in bulk and came up to 0.43 wt% of compost dry weight. Finally, the liquid fertilizer produced during the anaerobic treatment contained several thousand BDP fragments < 500 µm per liter. Hence, our study questions, if currently available BDP are compatible with applications in areas of environmental relevance, such as fertilizer production.

## Introduction

Biodegradable plastics (BDP) are increasingly proposed as eco-friendly alternatives to commodity plastics for foils, wrappings and bags. One area where the utilization of BDP could be of significant benefit is the collection of organic household waste. Currently most collected household biowaste is contaminated by conventional plastic bags, presumably because a significant fraction of the population prefers, if at all, to collect its biowaste in such bags. However, conventional plastics are not supposed to enter a biowaste treatment plant, since they will not degrade. In consequence they have to be removed as completely as possible from the incoming biowaste by elaborate sorting procedures, which incidentally also leads to significant losses of degradable organic material. Since the biogas (electricity, heat) and fertilizer produced from that material create the revenues, while the refuse has to be disposed at considerably costs, any such loss is not in the interest of the plant operators. In spite of the elaborate preparation, the entry of plastics into biowaste treatment plants cannot be completely prevented and strict regulation have been introduced inter alia in regard to the maximum amount of plastic allowed, e.g. in certified compost of high quality, such as < 0.1 wt% according to §3, 4b, DüMV and §3, 4c, DüMV. For reasons of practicability, only plastic fragments > 2 mm are counted for the quantification of the contamination, a limit which is expected to be lowered to fragments > 1 mm in the near future. In this situation, compostable plastic bags are seen as an attractive option, in particular since the conditions during technical biowaste treatment by composting should be ideal for their breakdown and dedicated bags for the purpose of household biowaste collection have appeared in supermarkets. Admittedly, not all adverse effects of foils and bags in biowaste treatments plants would automatically be resolved through the introduction of biodegradable bags. Operators have been known to fear for their machinery, in particular during anaerobic digestion, where biodegradable materials are not expected to disintegrate to a significant degree. However, much in this regard depends on the actual operation conditions. Plants with active mixing may face more difficulties than box plants.

A typical definition for biodegradability is given in European Norm EN 13432 (Requirements for packaging recoverable through composting and biodegradation—Test scheme and evaluation criteria for the final acceptance of packaging^1^), which states that a material is biodegradable, if it is converted (‘mineralized’) by microbial activity in the presence of oxygen into CO_2_, water, mineral salts, and biomass or in the absence of oxygen into methane, CO_2_, water, mineral salts, and biomass. While the definition is clear, actual biodegradation is typically estimated in a non-specific manner through a comparison of the CO_2_ produced by an aerobic standard culture in the presence of the test material compared to a culture without as well as a culture containing similar amounts of a natural biodegradable material such as cellulose. Under these circumstances nothing is learned about the mechanism of breakdown of the biodegradable material, in particular, if a significant part of it remains as micro- and nanoplastics, i.e. particles, which are considered to have considerable impact on environmental and human health^2^. Moreover, current biodegradable/compostable materials are not certified for disintegration under anaerobic conditions. In addition, the term compostable is used in the context of biodegradable plastics. EN 13432 defines a material as compostable, if 90 wt% of the material is fragmented (disintegrated) into particles < 2 mm, i.e. below the limit at which particles “count”, after twelve weeks of standardized composting and fully mineralized by 90 wt% within 6 months. The remaining 10 wt% may be transformed into biomass or simply be fragmented into microplastic. In addition, a compostable material may not bring heavy metals or introduce ecotoxic effects in the final compost.

Studies investigating the fate of BDP under realistic conditions, i.e., in technical systems for organic waste management (composting and biogas plants), are still rare, in particular in regard to fragments < 2 mm. A recent study by members of our group found that composts and fertilizers from biowaste treatment plants are a path of entry into the environment for microplastic^3^, but BDP was not considered in this case. Since then, a few studies on BDP in technical biowaste treatment and composting plants have appeared in trade journals^4–6^. However, these considered only residual fragments > 2 mm, which, according to these studies, were no longer in evidence after the composts had been conditioned by the customary sieving steps. In one case, foils certified as biodegradable were purposely introduced in controlled amounts into the digestion/composting process, and again no plastic fragments were visible in the finished—sieved—compost^6^. The size fraction < 2 mm was not considered in any of these studies.

Finally, the degradation of BDP in the environment has been studied. Admittedly, the certification of a material as biodegradable/compostable concerns the behavior of said material under composting conditions rather than a possible environmental impact, e.g. after littering. However, these environmental studies are highly relevant in regard to any residual BDP released into the environment with the composts. For instance, degradation in fresh and salt water, has for some BDP been less efficient than one would expect for a truly biodegradable material^7^. Physical properties seem to play a role, as some studies have shown a significant impact of a BDP’s crystallinity on its susceptibility to enzymatic depolymerization^8,9^. For microbial digestion under both aerobic^10^ and anaerobic^9^ conditions, the polyester PHBV (poly(hydroxybutyrate-cohydroxyvalerate) in the semicrystalline state was found to degrade more slowly than the corresponding amorphous material. Studies on the use of biodegradable foils for agricultural purposes^11–13^ show that BDP can persist for several years in the environment, while the question of whether they are indeed finally mineralized or merely disintegrated into yet smaller fragments under environmental conditions is not fully resolved.

Compostable materials are designed for disintegration/mineralization though composting. Technical composting plants provide optimal conditions for biodegradation, both in terms of the process conditions (temperature, intensive aeration) and the metabolic activity of the specialized microbial communities found therein. If mineralization is incomplete under these circumstances, the remaining material is released into the environment, where it may persist for an unknown time, with putatively all the negative consequences already known for commodity plastics^14,15^. The aim of this study was therefore, to determine to what extent residues of BDP can be found in the fertilizers (compost, liquid fertilizer) produced by organic waste treatment plants and thereby contribute to an ongoing discussion of whether the currently available BDP are already suited to replace conventional plastics in environmentally sensitive areas.

## Results and discussion

### Choice of biowaste treatment plants and sample identifiers

Compost samples were collected from four central municipal biowaste treatment plants (denominated as #1 to #4) in Baden-Wurttemberg, Germany (Table 1). All plants used a state-of-the-art two-stage biowaste treatment process comprising of (a) anaerobic digestion/biogas production and (b) subsequent composting of the solid digestate to produce a high-quality mature compost sold for direct use as fertilizer in agriculture. The composts were regularly analyzed by an independent laboratory for quality and residual contamination and consistently fulfilled the quality requirements of the label RAL-GZ 251 Gütezeichen Kompost of the German Bundesgütegemeinschaft Kompost e.V. (www.gz-kompost.de). Plants #1 and #3 produce in addition a liquid fertilizer, which is separated from the solid digestate at the end of stage a) by press filtration and which is also intended for direct use on agricultural soil (replacement of liquid manure). In case of plants #1, #3, and #4 up to 25 wt% of shrub/tree cuttings were added to the solid digestate for composting. All plants used sieving (typically with a 12 or a 20 mm mesh) at the end of the process to assure the necessary purity of their finished composts. Whenever technically possible, we as well took samples of the pre-compost immediately before this final sieving step to evaluate its contribution to the removal of residual BPD fragments. For analysis, composts were passed consecutively through two sieves with mesh sizes of 5 mm and 1 mm, yielding two fragment preparations for IR-analysis namely a > 5 mm fraction corresponding to the contamination by residual “macroplastic” (5 mm is a commonly used upper size limit for “microplastic”, anything larger is macroplastic) and a 1–5 mm fraction corresponding to the regulatory relevant residual contamination by microplastic. The lower limit of 1 mm rather than 2 mm was chosen in anticipation of the expected changes in regulation, where the replacement of the 2 mm limit by a 1 mm limit is imminent.

**Table 1:** Technical data of the investigated plants and incidence of BDP fragments in the sampled composts.

	Plant #1		Plant #2		Plant #3						Plant #4			
Compost sample type	Finished		Finished		Pre			Finished			Pre		Finished	
Biowaste preparation	Shredder, sieving (80 mm)		None		Shredder						Cross-flow shredder, sieving (2 lines: biowaste 60 mm, shrub/tree cuttings 80 mm)			
Anaerobic digestion	Plug flow 55 °C average 21 days		Box fermenter 40 °C average 40 days		Plug flow 55 °C average 21 days						Plug flow average 21 days^†^			
Composting	Up to 5 weeks		At least 5.5 weeks		Up to 9 weeks						6 weeks			
Final sieving step	12 mm		20 mm					Yes^b^					12 mm	
Products	Compost and liquid fertilizer^a^		Only compost		Compost and liquid fertilizer^a^						Only compost			
Fragment size (mm)	> 5	1–5	> 5	1–5	> 5	1–5		> 5		1–5	> 5	1–5	> 5	1–5
Number of BDP fragments per kg of compost^c^	16	18	–	19	29	3		–		2	4	–	–	–

^a^Part of the liquid fraction was returned into the fermenter for mashing the substrate. ^†^One part of the digestate is dried and composted, another part is mixed with fresh substrate and returned to the fermenter.

^b^No details available.

^c^Dry weight.

### Occurrence of plastic fragments > 1 mm in the sampled composts

**Figure 1:**
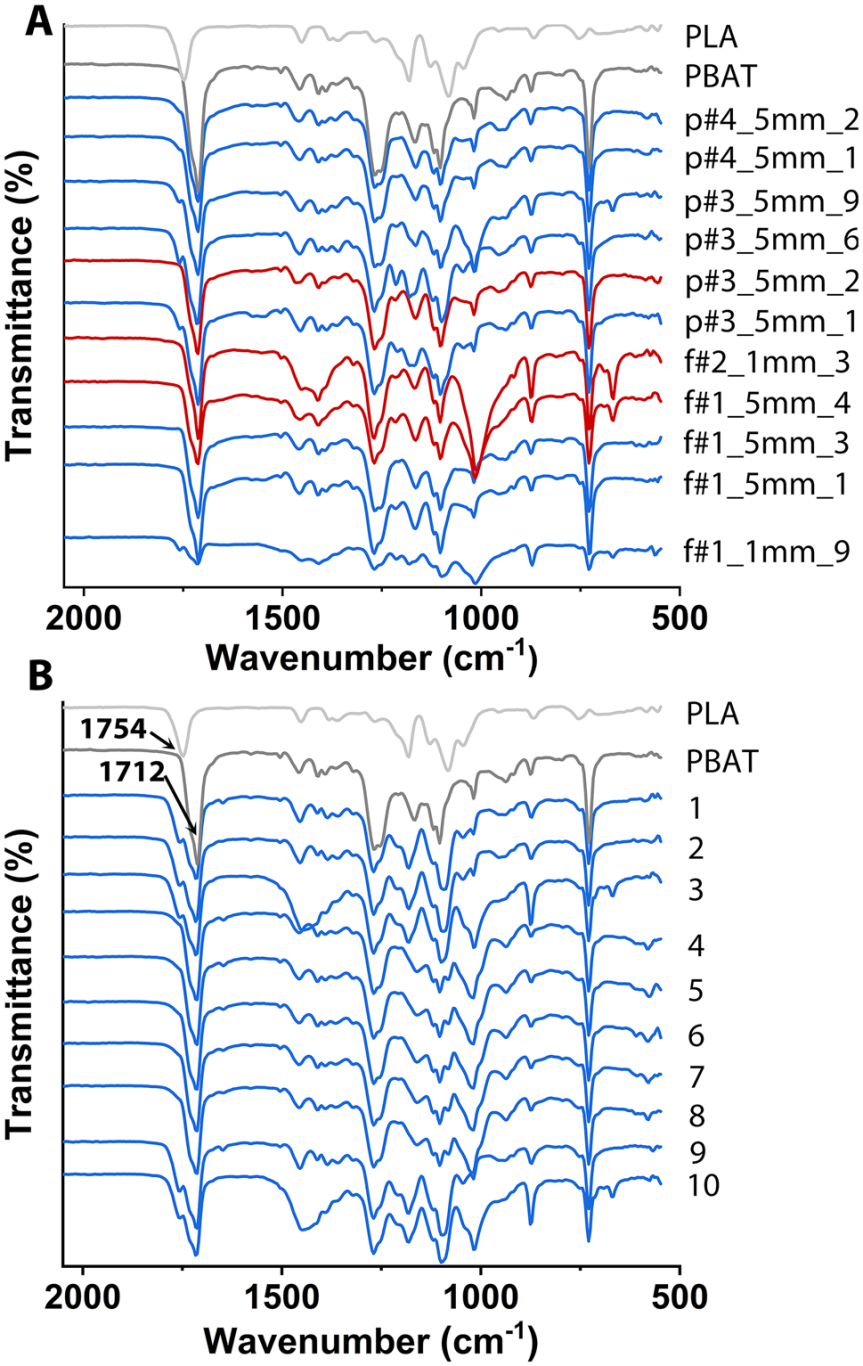
FTIR spectra of BDP fragments from composts and commercial bags. (**A**) BDP fragments recovered from the composts and (**B**) the commercial compostable bags. Fragments were coded as follows: p or f for pre-compost or finished compost, followed by the plant number (#1 to #4), an indication of the size fraction (> 5 mm or 1–5 mm) in which the fragment was found, and finally, the fragment number. Fragment F#1_5mm_4 therefore represents the 4th fragment collected in the > 5 mm size fraction from the finished compost of plant number 1. Bags were arbitrarily numbered 1–10, see Suppl Table S1 for supplier information. The spectra (in grey) of the reference materials for PLA and PBAT are given as basis for the interpretation. Spectra in red refer to test samples consisting only of PBAT, while those in blue indicate samples composed of PBAT/PLA mixtures.

Composting times of 5–9 weeks were used in the investigated plants (Table 1), which is shorter than the 12 weeks indicated in EN 13432 for the 90% disintegration of a compostable plastic material, but a realistic time span for state-of-the-art technical waste treatment. Since we were not in a position to estimate the quantity of BDP entering the plants, since for technical reasons we were unable to obtain a representative sample, we cannot say, whether any residual BDP detected by us in the finished composts was due to a yet incomplete disintegration process or whether it corresponds to the 10% material still permissible by EN 13432 even after the full composting step. However, in 7 out of the 12 sampled composts and pre-composts fragments with chemical signatures corresponding to the BDPs poly (lactic acid) (PLA) and poly (butylene-adipate-co-terephthalate) (PBAT) were identified in the > 5 mm and/or the 1–5 mm sieving fractions using FTIR analysis^3^ (Fig. 1; Table 1). All recovered fragments appeared to stem from foils, bags or packaging, since they were thin compared to their length and width (see Suppl Figure S1 for typical examples). Fragments with overlapping signatures, most likely PBAT/PLA mixtures or blends, were also found (see Suppl Figure S2 for the interpretation of the spectra). In addition, the recorded BDP fragment spectra (Fig. 1A) showed high similarity to the FTIR spectra of commercial compostable bags sold in the vicinity of the biowaste treatment plants (Fig. 1B), which together with the geometry of the recovered fragments led us to assuming that the majority of the BDP entered the biowaste in the form of such bags.

**Figure 2:**
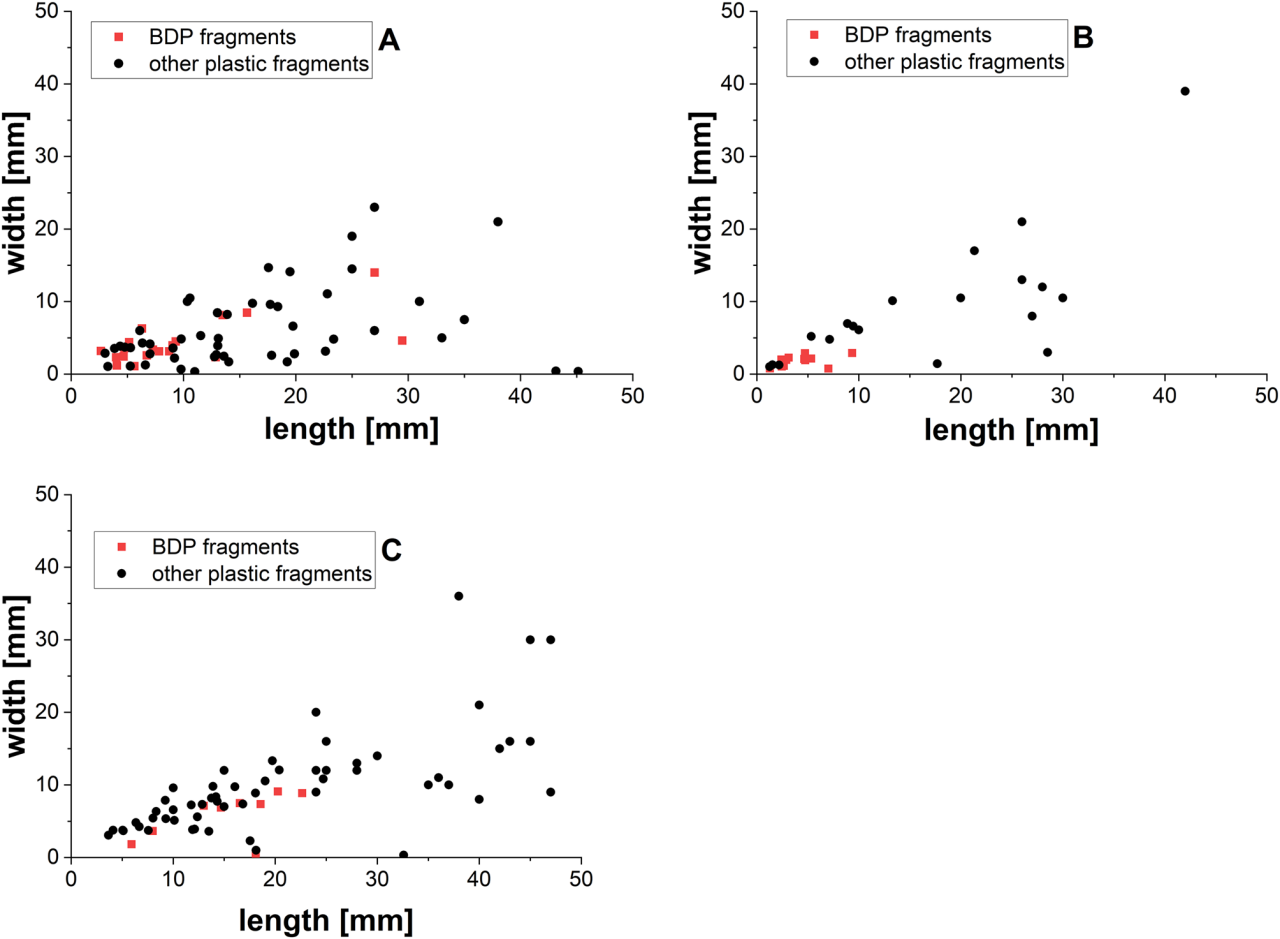
Size distribution of plastic fragments > 1 mm. (**A**) Fragments found in the finished compost from plant #1, (**B**) in the finished compost from plant #2, and (**C**) in the pre-compost from plant #3. For reasons of statistical relevance, only samples containing more than 20 BDP fragments per kg of compost were included in the analysis.

The BDP fragments were found alongside fragments of commodity plastics (mostly PE) in all cases. Finished composts tended to contain fewer and smaller fragments than the corresponding pre-composts. The final sieving of the pre-composts to prepare the finished composts hence appears to be quite effective in removing such fragments, in particular those from the > 5 mm size fraction (Table 1) and for that reason has become state-of-the-art in preparing quality composts (contamination by plastic fragments > 2 mm of less than 0.1 wt%). Given that the size of the fragments is a crucial factor regarding ecological risk, we analyzed the sizes (length Î width) of the BDP fragments in comparison to that of the plastic fragments with signatures of commodity plastics such as PE (Fig. 2). BDP fragments found in a given compost sample tended to be smaller than the fragments stemming from non-BDP materials, which may indicate that BDPs degrade faster or tend to disintegrate into tinier particles than commodity plastics. This may also explain why in the compost from plant #2, no BDP fragments were found in the particle fraction retained by the 5 mm sieve (> 5 mm fraction), while 19 such particles were found in the fraction then retained by the 1 mm sieve (1–5 mm fraction). Interestingly, plant #2 is the only one included in our study that uses no mechanical breakdown of the incoming biowaste. This reduces the mechanical stress on the incoming material. Mechanical stress can alter the properties of plastic foils such as the crystallinity whereby crystallinity has been shown to influence the biological degradation of BDP such as PLA^7^.

### Material characteristics of BDP fragments in comparison to those of commercial biodegradable bags

In order to verify whether the BDP fragments recovered from the composts differed from the compostable bags in any parameter with possible relevance for biodegradation and environmental impact^16^, the physico-chemical properties of bags and fragments were studied in detail. Since we wanted to have a maximum of information of the BDP fragments, size/weight was a limiting factor in selecting fragments for analysis. Fragments of at least 1 mg were required for the FT-IR analysis. 5 mg-fragments could be analyzed in addition by ^1^H-NMR, while the full set of analytics (FT-IR, ^1^H-NMR, and DSC) required at least 10 mg of sample.

**Figure 3:**
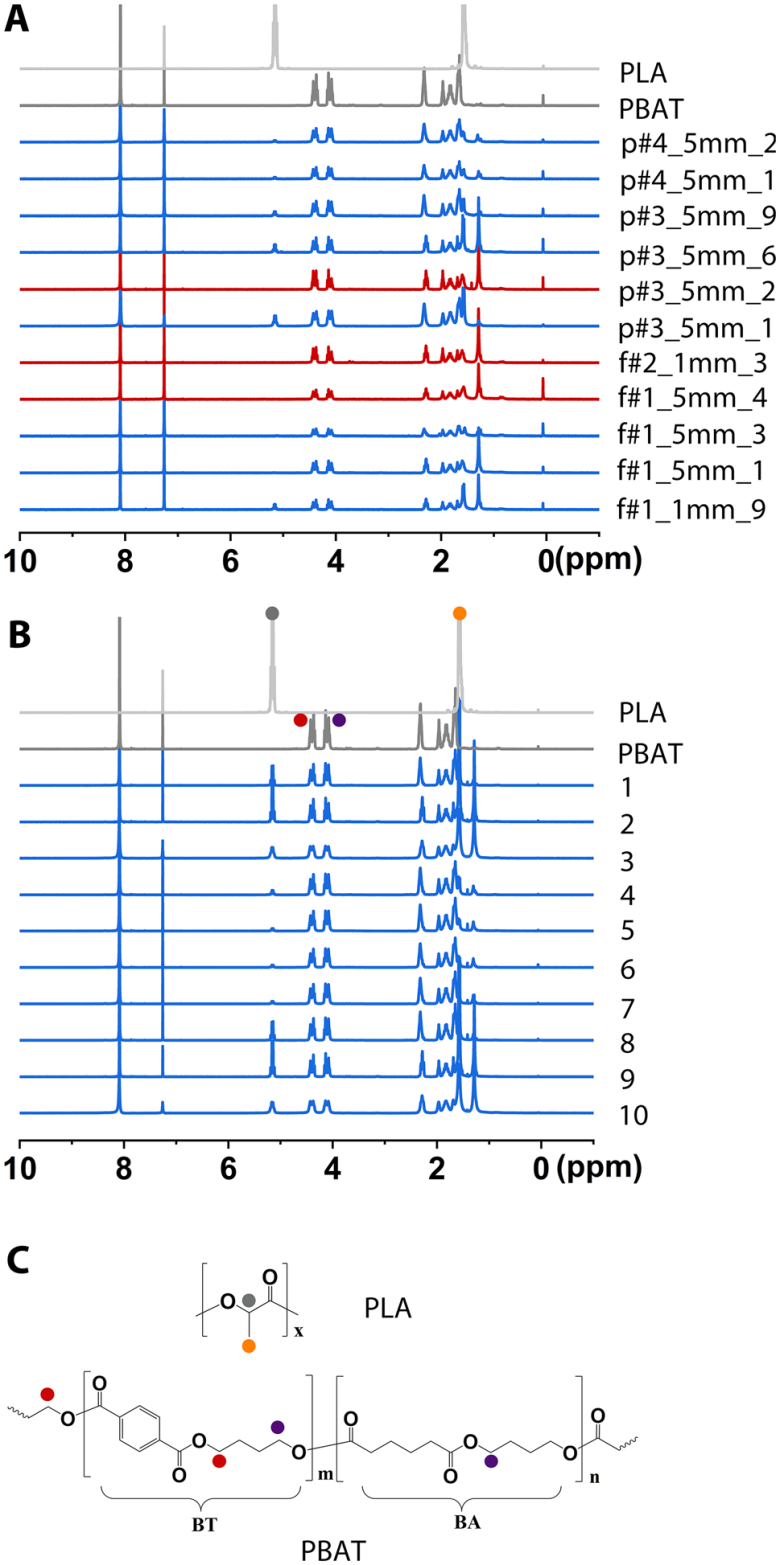
^1^H NMR spectra of BDP fragments from composts and commercial bags. (**A**) BDP fragments recovered from the composts and (**B**) the commercial compostable bags. Fragments were coded as follows: p or f for pre-compost or finished compost, followed by the plant number (#1 to #4), an indication of the size fraction (> 5 mm or 1–5 mm) in which the fragment was found, and finally, the fragment number. Bags were arbitrarily numbered 1–10, see Suppl Table S1 for supplier information. The spectra (in grey) of the reference materials for PLA and PBAT are given as basis for the interpretation. Spectra in red refer to test samples consisting only of PBAT, while those in blue indicate samples composed of PBAT/PLA mixtures. (**C**) Chemical structures of PLA and PBAT, chemical shifts of the protons are assigned as indicated in the reference spectra in (**B**).

For insight into the chemical composition, ^1^H-NMR spectra of the commercial bags and all suitable BDP fragments were compared (Fig. 3). In case of material mixtures and blends, the ^1^H-NMR analysis allows quantification of the PBAT/PLA weight ratio in the materials and also of the ratio of the butylene terephthalate (BT) and butylene adipate (BA) units in the involved PBAT polyesters.

The ^1^H-NMR spectra corroborate the FTIR measurements in that all investigated commercial bags were made from PBAT/PLA mixtures of varied composition (Table 2). By comparison, some of the fragments, for instance, f#1_5mm_4, appeared to consist of only PBAT. Other fragments, e.g., f#1_1mm_9, were mixtures of PLA and PBAT (Table 2). However, even in the case of PBAT/PLA mixtures, the average PBAT content tended to be higher in the fragments than in the bags, while the BT/BA monomer ratio in the respective PBATs, was also significantly higher in the fragments than in the bags. If we assume the fragments to stem from similar compostable bags as the ones included in our comparison, this would mean that during composting of such a bag, the PLA degrades more quickly than the PBAT, whereas within a given PBAT polyester, the BA unit is more easily degraded than the BT unit. Evidence can indeed be found in the pertinent literature that PLA has faster biodegradation kinetics than PBAT, while BT is more resistant to mineralization than BA^17,18^.

**Table 2:** Composition of commercial compostable bags and BDP fragments recovered from the composts as analyzed by ^1^H-NMR.

Sample	PBAT (wt%)	PLA (wt%)	BT/BA-ratio in the PBAT	
Bag No.1	79.3	20.7	0.86	Average PBAT (wt%): 80.8 ± 12.1 Average BT/BA ratios: 0.91 ± 0.06
Bag No.2	66.1	33.9	0.96	
Bag No.3	68.8	31.2	1.01	
Bag No.4	92.0	8.0	0.87	
Bag No.5	95.4	4.6	0.87	
Bag No.6	95.4	4.6	0.86	
Bag No.7	95.6	4.5	0.88	
Bag No.8	79.3	20.7	0.85	
Bag No.9	66.1	33.9	0.94	
Bag No.10	70.2	29.8	1.01	
f#1_1mm_9	82.5	17.5	0.99	Average PBAT (wt%): 92.8 ± 7.9 Average BT/BA ratios: 1.02 ± 0.12
f#1_5mm_1	99.2	0.8	1.00	
f#1_5mm_3	97.4	2.6	1.02	
f#1_5mm_4	100	–	1.02	
f#2_1mm_3	100	–	1.15	
p#3_5mm_1	77.9	22.1	0.93	
p#3_5mm_2	100	–	1.33	
p#3_5mm_6	82.1	17.9	0.99	
p#3_5mm_9	89.9	10.1	0.95	
p#4_5mm_1	96.2	3.8	0.94	
p#4_5mm_2	95.6	4.4	0.90	

BT/BA-ratio in the PBAT: BT, butylene terephthalate; BA, butylene adipate, molar ratio of the two monomeric units found in the co-polyester PBAT. Fragments were coded as follows: p or f for pre-compost or finished compost, followed by the plant number (#1 to #4), an indication of the size fraction (> 5 mm or 1–5 mm) in which the fragment was found, and finally, the fragment number.

**Figure 4:**
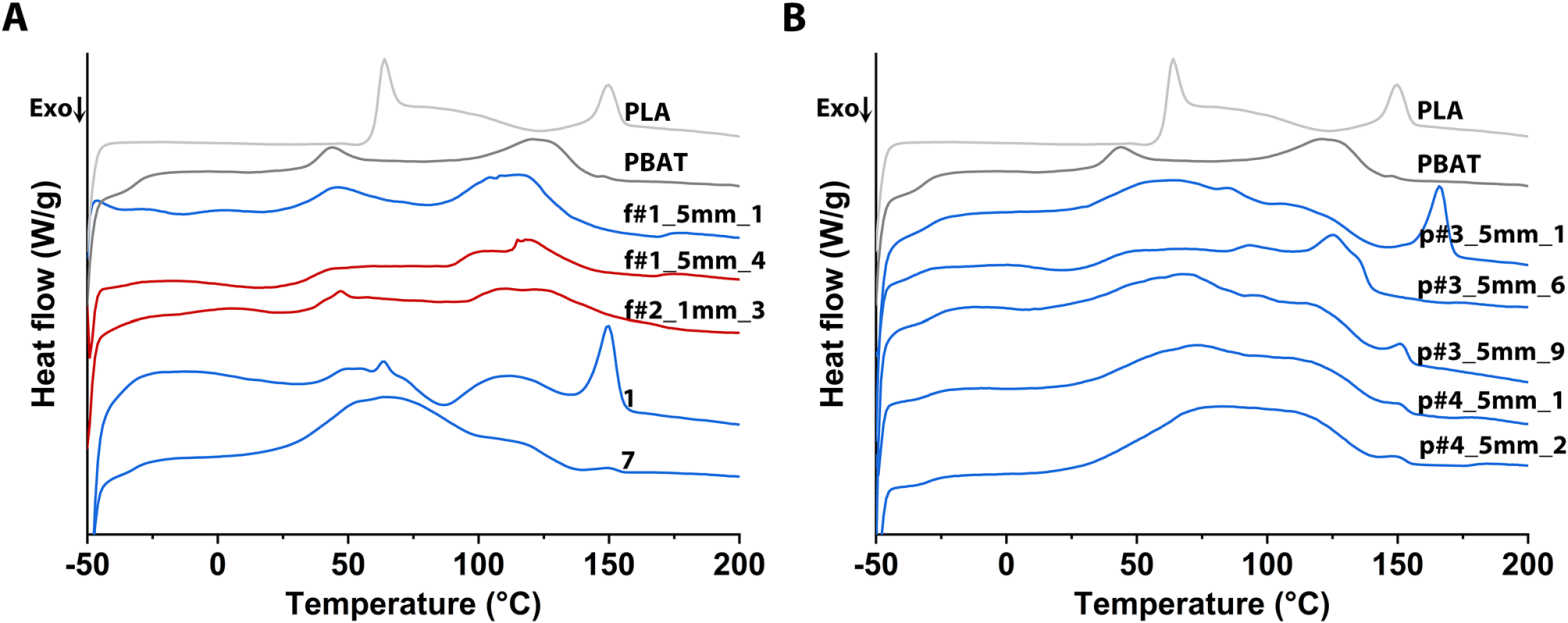
DSC curves of BDP fragments and compostable bags #1 and #7. Curves for the reference materials (in grey) for PLA and PBAT are given for comparison. Curves were recorded during the first heating run (temperature range: − 50 °C to 200 °C, heating rate: 10 °C min^−1^). (**A**) and (**B**) curves in red refer to test samples consisting only of PBAT, while those in blue indicate samples composed of PBAT/PLA mixtures. Fragments were coded as follows: p or f for pre-compost or finished compost, followed by the plant number (#1 to #4), an indication of the size fraction (> 5 mm or 1–5 mm) in which the fragment was found, and finally, the fragment number.

Next, differential scanning calorimetry (DSC) was used to analyze fragments compared to commercial bags in regard to the presence of amorphous vs. crystalline domains, a parameter expected to affect biodegradation kinetics and therefore the putative environmental impact of the produced microplastic^16^ upon release into the environment with the composts. Whereas amorphous domains show glass transition, crystalline domains show melting, both of which can be discerned by the respective phase transition enthalpy in the DSC curves (Fig. 4).

The curve for the reference PBAT shows a glass transition temperature (Tg) of − 29 °C and a broad melting range between 100 and 140 °C for the crystalline domains, while that of the PLA reference shows a glass transition temperature of 58 °C and a narrower melting peak between 144 °C and 162 °C. The curve for commercial bag #1, which had a comparatively high PLA content, shows a pronounced melting peak in the expected range; the same is the case for fragment p#3_5mm_1 and to a lesser extent for fragment p#3_5mm_9, two fragments, which also have high PLA contents. The DSC curves of the other fragments and bag #1 are undefined in comparison, which is due to their high PBAT content. According to the DSC curves, most of the investigated materials are semicrystalline, i.e., contain both amorphous (glass transition) and crystalline (melting) domains. However, the DCS data alone allow only a qualitative discussion of the differences between fragments and bags.

**Figure 5:**
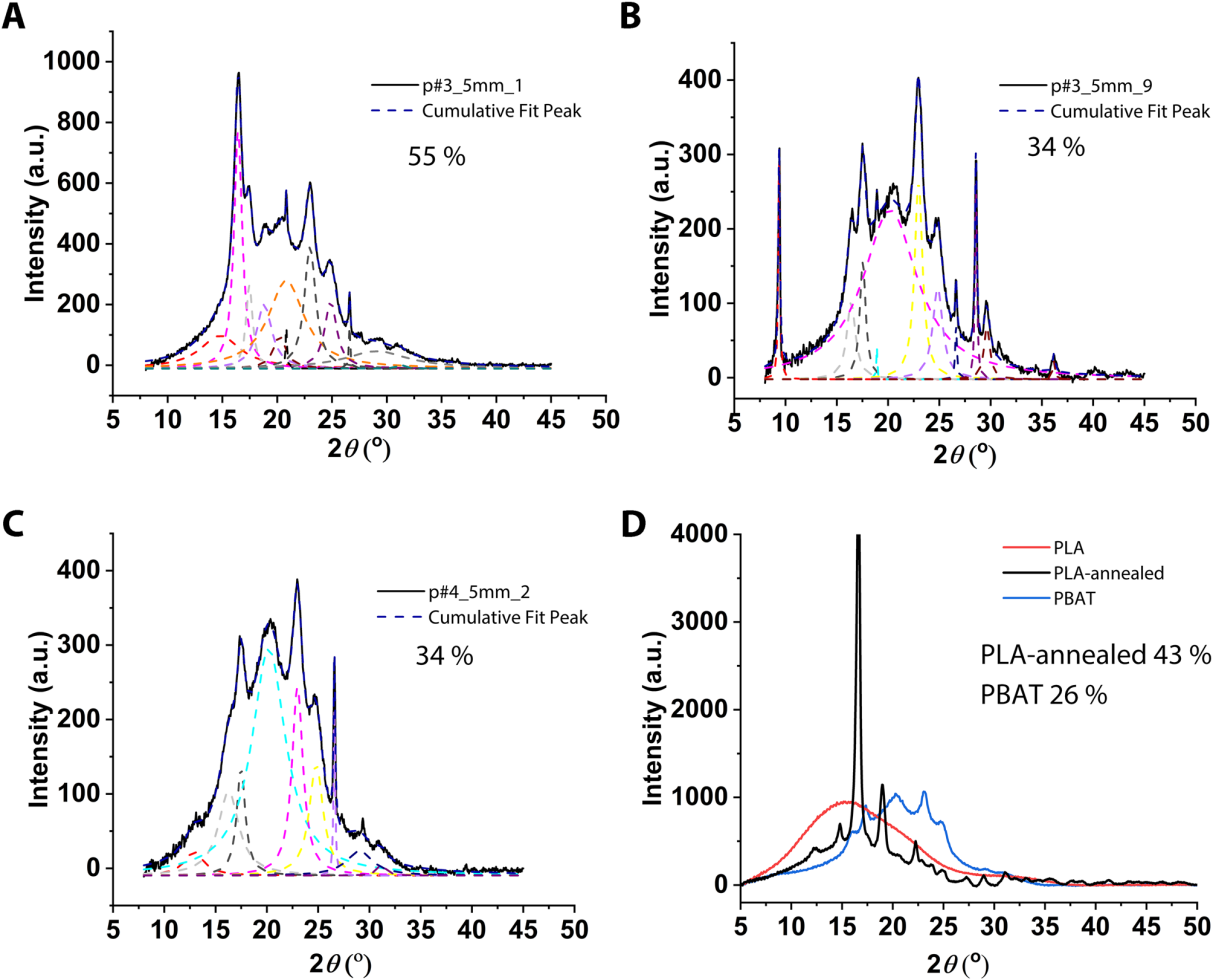
WAXS curves with Lorenz fitting for (**A**) fragment p#3_5mm_1, (**B**) fragment p#3_5mm_9, and (**C**) fragment p#4_5mm_2. (**D**) WAXS curves for foils produced from the PBAT and PLA reference materials; the percent values indicate the crystallinity. The dash lines are the fitting peak curves for the XRD spectrum. Crystallinity can be obtained by dividing the integration area of the fitted peaks by the integration area of the entire spectrum. Fragments were coded as follows: p or f for pre-compost or finished compost, followed by the plant number (#1 to #4), an indication of the size fraction (> 5 mm or 1–5 mm) in which the fragment was found, and finally, the fragment number.

To obtain quantitative data on the crystallinity differences, wide angle X-ray scattering (WAXS) spectra were recorded. WAXS requires fragments at least 3 cm long, which restricted the number of fragment samples to three, all of which were found in pre-compost samples. The corresponding curves are shown in Fig. 5A–C. The spectra of the commercial biodegradable bags are shown in Suppl Figure S3. Foils were in addition prepared by heat pressing from the reference materials for PLA and PBAT in order to include them into the WAXS measurements (Fig. 5D). While the foils produced from the PBAT reference material produced crystallinity peaks at 16.2°, 17.3°, 20.4°, 23.2°, and 24.8°, the foil prepared from the PLA reference material showed only an amorphous halo at 15.5° and 31.5°, which is in accordance with values published in the literature^19^. A more pronounced crystallinity peak was obtained in the case of an additionally annealed PLA foil.

In case of the fragments and bags, the peaks of PLA and PBAT overlapped to some extent in the WAXS spectra, but by conducting Lorenz fitting using Origin software, the overall crystallinity could be calculated as follows:

$$\chi = { 1}00\% \, *{\text{ Aa}}/\left( {{\text{Aa }} + {\text{ Ac}}} \right)$$

where χ is the crystallinity and Aa and Ac represent the areas of the amorphous and crystalline peaks.

Using this equation, crystallinities of 55% (fragments p#3_5mm_1), 34% (p#3_5mm_9), and 34% (p#4_5mm_2) were calculated for the fragments. The foils prepared in house for the reference materials had similar crystallinities (43% in case of the annealed PLA foil and 26% of the PBAT foil), while the simple PLA foil was amorphous. By comparison, for eight of the commercial bags, crystallinities in the range from 1% to 7% were calculated, whereas these values were 14% and 15% for the remaining two bag types (Suppl Figure S3).

The high crystallinity of the larger fragments recovered from the pre-compost samples suggests that crystalline domains of BDP materials may indeed disintegrate more slowly than the amorphous ones, as prior studies on microbial biodegradation have suggested^7,8^. Admittedly, such large fragments *per se* would not enter the environment, since the final sieving step used to prepare the finished composts is quite efficient at removing them. However, it is tempting to extrapolate that residual BDP in general are remnants of the more crystal domains of the original material, even though experimental proof of this assumption is at present not possible. 10 wt% of a BDP bag is allowed to remain after standard composting. It is usually assumed that any such residues continue to degrade with comparable speed. However, should these residues correspond to the more crystalline domains, rather than degrading with similar speed as the bulk material, the more crystalline fragments can be expected to persist for a much longer and at present unpredictable length of time in the environment, e.g. when applied to the soil with the composts; in particular, when they are also enriched in PBAT and BT units as suggested by our analysis of the chemical composition. Data from the use of biodegradable foils in agriculture show that the degradation in the environment may take years^20^. Altogether this may have unforeseen economic and environmental consequences, especially when considering the high fraction of BDP fragments < 5 mm. Putative consequences include changes in soil properties, the soil microbiome and therefore in plant performance^21^, a factor indispensable for worldwide nutrition.

### Residues of BDP fragments < 1 mm in liquid fertilizer and in composts

In addition to the composts, plants #1 and #3 produce a so-called liquid fertilizer (LF). LF is applied directly to agricultural soil without further treatment. No plastic fragments > 1 mm were found in the collected LF samples. This is hardly surprising, given that the LF is produced by press filtration of the digestate after the anaerobic stage. Such a filtration step can be expected to retain fragments > 1 mm in the produced filter cake, which goes into the composting step, leaving the filtrate, i.e. the LF, essentially free of such particles. Anaerobic digestion is currently not assumed to contribute significantly to the degradation of BDP^17,22^, but the process conditions (mixing, pumping) may promote breakdown of larger fragments, particularly when additives such as plasticizers^23^ leach out of the material.

Since the residual solids content of the LF is low (plant #1: 8.6 wt%, plant #3: 5.8 wt%), a combination of enzymatic-oxidative treatment and µFTIR imaging originally developed for environmental samples from aqueous systems^24,25^ could be adapted for the analysis (size and chemical signature) of particles in the LF down to a size of 10 µm. The corresponding data are compiled in Table 3. In all cases, residual fragments from PBAT-based polymers represented the dominant plastic fraction in the investigated samples; i.e. approximately 53% of all plastic particles in the LF from plant #1 (11,520 BDP particles per liter) and 65% in the case of plant #3 (12,480 BDP particles per liter). Liquid manure is applied several times a year to fields at a concentration of 2–3 L m^−2^. According to our analysis > 20,000 BDP microparticles of a size ranging from 10 µm to 500 µm enter each m^2^ of agricultural soil whenever LF is applied on agricultural surfaces.

**Table 3:** Microplastic fragments (BDP/all) found per liter of liquid fertilizer.

Plant	11–22 µm	22–100 µm	100–300 µm	300–500 µm	500–1000 µm	%
#1	0/0	3840/8960	6400/8960	1280/2560	0/1280	53
#3	1280/1920	7040/10,880	3840/6080	320/320	0/0	65

%: Percentage of BDP fragments within all recovered plastic fragments.

Due to the complexity of the matrix, a similar analysis of individual plastic fragments < 1 mm was not possible in case of the composts. These were instead subjected to an organic solvent extraction after removal of all fragments > 1 mm. Six compost samples representing the more contaminated ones based on the content of fragments > 1 mm, namely, f#1, f#2, p#3, f#3, p#4 and f#4 (nomenclature: f or p for finished or pre-compost, followed by plant number), were extracted with a 90/10 vol% chloroform/methanol mixture. The amounts of PBAT and PLA in the obtained extracts were then quantified via ^1^H-NMR (Table 4). Briefly, the intensity of characteristic signals in the extract spectra of the compost samples (see Suppl Figure S4) were compared to peak intensities produced by calibration standards of the pure polymer dissolved at a known concentration in the chloroform/methanol. All samples and standards were normalized using the 1,2-dichloroethan signal at 3.73 ppm as internal standard. See also Suppl Figure S5 for an exemplification of the quantification of the PBAT/PLA ratios. Based on the amounts of PBAT and PLA extracted from a known amount of compost, the total mass concentration (wt% dry weight) of these polymers in the composts was calculated.

**Table 4:** Evidence of PBAT and PLA residues caused by fragments < 1 mm in the composts.

	f#1	f#2	p#3	f#3	p#4	f#4
Dry weight [%]	45.9	64.9	57.5	57.4	39.7	51.6
M_c_ [g]	100	100	65	54	100	100
M_e_ [g]	0.78	1.41	0.51	0.45	1.14	0.68
M_0_ [mg]	14.0	14.8	14.8	12.2	15.3	13.5
M_STD_ [mg]	9.1	8.9	9.5	12.0	8.6	15.2
M_PLA_ [mg]	1.488	1.259	1.300	0.369	2.645	1.788
M_PBAT_ [mg]	1.228	3.283	1.102	0.381	0.370	0.148
P_PLA_ [%]	10.6	8.5	8.8	3.0	17.3	13.2
P_PBAT_ [%]	8.8	22.2	7.4	3.1	2.4	1.1
C_PLA_ [ppm]	827	1199	690	250	1972	898
C_PBAT_ [ppm]	686	3130	581	258	274	75
A_PLA_ [m^2^]	29.00	42.04	24.19	8.77	69.14	31.49
A_PBAT_ [m^2^]	23.67	108.01	20.05	8.90	9.45	2.59

M_c_: mass of dry compost subjected to extraction; M_e_: mass extracted from compost sample; M_0_: mass of material used for ^1^H NMR; M_STD_: mass internal standard used for ^1^H NMR; M_PLA_, M_PBAT_: masses of PLA and PBAT in M_0_; P_PLA_, P_PBAT_: wt% of PLA and PBAT in M_e_; C_PLA_, C_PBAT_: mass concentration of PLA and PBAT in M_c_; A_PLA_ and A_PBAT_: calculate surface area covered by PLA and PBAT in 1 ton of dry compost.

Compost samples contained between 0.5 and 1.5 wt% extractable material out of which between 6 wt% and 30 wt% were made up of the biodegradable polymers PLA and PBAT. In consequence, the compost samples contained between 0.05 and 0.43 wt% PLA and/or PBAT < 1mm per unit dry weight. This is in the same order of magnitude and even above the current limit (0.1 wt%) for certified composts in regard to the contamination with plastic fragments^26^ > 2 mm. Moreover, residues of PBAT and PLA were found in all investigated compost samples, including the finished compost from plant #4, which had shown no contamination by larger BPD fragments (Table 1). The pre-compost from that plant had shown a few contaminating BDP fragments in the > 5 mm fraction. However, in regard to the fragments < 1 mm, the composts from plant #4 showed a similar incidence, at least for PLA, as the finished compost samples from the other plants (Table 4).

Since the material was extracted and quantified in solution, no direct information regarding the original dimension of the fragments < 1 mm could be derived. However, if we assume a similar thickness as for the larger fragments or commercial bags (17–25 µm) together with densities of 1240 kg m^−3^ (PLA) and 1260 kg m^−3^ (PBAT) as measured for the corresponding reference materials, the particles < 1 mm found in one ton of these composts would, when placed side by side, cover an area between 17 and 150 m^2^ (see values A_PLA_ and A_PBAT_ in Table 4). Therefore, if 10 tons of such compost were to be distributed over 1 ha (10,000 m^2^) of agricultural surface, which is not unreasonable^27^, the added plastic particles < 1 mm combined would theoretically cover up to 15% of this area. Taken together with the data on larger BDP fragments and on commodity plastics, environmental contamination via composts may be much higher than previously thought^3^.

Given that our results show that predominantly tiny BDP fragments (microplastic) enter the environment via compost and LF, a possible impact on environmental and finally human health and nutrition is indicated. Polymer particles in the micron- and nanometer range have already been shown to be more toxic than larger ones^2,28,29^. In addition, the coverage with an ecocorona^30^, that will certainly take place during digestion/composting, facilitates the internalization into cells^31^ and therefore increases the risk associated with the ingestion of microplastic, e.g. by soil macrofauna^32^. Finally, the higher crystallinity and therefore higher resistance to further biodegradation extends the period of bioavailability of BDP microparticles with all the above-mentioned consequences. Whether BDP fragments with higher crystallinity or a higher BA unit within the PBAT co-polyester also induce stronger toxic effects remains to be investigated. In this view, the mechanisms and kinetics of BDP breakdown under conditions of industrial biowaste treatment, but also in soils used for food and feed production, should be investigated in more detail, before the widespread use of the currently available biodegradable materials as presumably environmentally friendly alternatives to conventional plastics is advocated.

## Materials and methods

### Materials

If not otherwise indicated, suppliers for chemicals were Th. Geyer (Renningen, Germany) and Sigma-Aldrich (Taufkirchen, Gemany). Ultrapure water was produced with an Elga-Veolia-Purelab (Flex2) unit, while ‘Millipore-water’ came from a Millipore-Synergy-UV-system (Type 1). Compostable bags (10 different brands) designated for the collection of organic waste by the supplier were bought from different local supermarkets (Table S1). Polymer reference materials for BDP were: PLA (batch no.: GH0728B133, commercial name: Ingeo Biopolymer 4043D, supplier: NatureWork, Minnetonka, MN) and PBAT (batch no.: 95010016KO, commercial name: Ecoflex F Blend C1200, supplier: BASF). Protease A-01 (activity: > 1.100 U mL^−1^), Pektinase L-40 (activity: > 900 U mL^−1^, Exo PGA, > 300 U mL^−1^ Endo PGA, > 300 U mL^−1^ Pektinesterase), and Cellulase TXL (activity: > 30 U mL^−1^) were from ASA Spezialenzyme GmbH (Wolfenbüttel, Germany), Viscozyme L (activity: > 100 FBG U g^−1^) was from Novozymes A/S (Bagsværd, Denmark).

### Sampling of composts and liquid fertilizer

Bulk samples were taken from composts according to the guidelines of the German Association for Quality Compost^26^. A slight modification to the standard procedure was introduced to avoid contacts of the compost samples with the plastic foil recommended in the standard protocol for sample mixing. Instead, the individual aliquots obtained from a given compost heap were pooled, mixed and stratified directly on the concrete floor (after a ‘washing’ step with compost from the same heap). To obtain a representative sample, the interior of the heap was made accessible using a wheel loader. Then, individual samples were taken at evenly dispersed points. The number and volume of the individual samples depended on the volume and grain size of the compost pile. For example, in the case of 100 m^3^ of a compost with grain sizes of 2–20 mm, 16 individual samples (1 L each) were taken, and 4 mixed samples (2 L each) were created at minimum. Whenever possible, samples of both the pre-compost (before the final sieving step) and the finished compost were taken. Pre-compost sample volumes were determined based on the volume required for the corresponding finished compost samples. Pre-compost and finished compost were sampled at the same time. Consequently, they represented different processing batches. Sample aliquots were transferred to 3 L Fido jars (Bormioli Rocco, Fidenza, Italy) for transport. If immediate analysis was not possible, samples were stored at 4 °C in the glass vessels. Samples of liquid fertilizer (~ 6 L) were collected from the outlet of the storage tanks also into glass vessels. The first few liters of liquid fertilizer were discarded to rinse the outlet pipe and ensure that representative samples were obtained. If necessary, liquid fertilizer samples were also stored at 4 °C. Backup samples of approximately 1 L were taken for all samples and stored at − 20 °C. Glass vessels for transport, storage or backup samples were rinsed in advance with Millipore water.

### Analysis of plastic fragments in the composts

A significant concern during the analysis in particular of microplastic particles in environmental samples is the possible contamination of samples with microplastic particles from the ambient air, clothing, laboratory tools or reagents used during sample preparation. In order to avoid contamination, precautionary measures were taken. Cotton lab coats were worn throughout. Unless direct handling was necessary, samples were covered with a glass or aluminum foil lid. Sample processing took place in a laminar-flow-box to prevent airborne particles from falling into the sample. All laboratory tools used were made of glass, metal or polytetrafluorethylene (PTFE), a polymer which is rare in environmental samples and is excluded from the analysis. All required solutions and the deionized water used to prepare them were filtered through 0.2 µm pore membranes (mixed cellulose ester membrane, diameter 47 mm, Whatman ME 24, Merck KGaA) before use. Enzyme solutions were filtered through 0.45 µm membranes (regenerated cellulose membrane, diameter 100 mm, Whatman RC 55, Merck KGaA) and stored in glass bottles with glass caps, ready for use. All laboratory equipment was thoroughly rinsed with filtered deionized water, 35% ethanol, and again filtered water before use and in between steps to avoid cross contamination. Blanks undergoing the same treatment as the environmental samples were used in order to detect possible contamination in the laboratory.

Prior to analysis, compost samples were filled into a rectangular metal form (790 mm × 510 mm × 150 mm), homogenized with a metal shovel and quartered. From two quarters (bottom right, top left), samples were taken for the investigation of the plastic content. Samples for the determination of the dry weight (DW) were taken from the bottom left quarter, while backup samples (1 L) were taken from the top right quarter. For the determination of the DW 100 mL sample aliquots were weighed into 250 mL Schott-Duran beakers and dried at 105 °C (oven: Memmert UM 500, Memmert, Schwabach, Germany) for at least 24 h. Afterwards, the beakers were allowed to cool to room temperature in a desiccator and the DW determined by weighing the beakers again.

For the recovery of the fragments > 1 mm, approximately 3 L of the compost sample was weighed and evenly distributed into 6 glass vessels (capacity 3 L each). The material was suspended in 2.5 L of water and first sieved with a mesh size of 5 mm (yielding fraction > 5 mm). All particles retained by the sieve were collected with tweezers and transferred to the system for ATR-FTIR analysis, see below, while the material passing the sieve was sieved again at 1 mm, followed again by collection of the retained particles (yielding fraction 1–5 mm), which were subsequently also analyzed by ATR-FTIR. Sieves were from Retsch GmbH (Haan, Germany; test sieve, IS 3310-1; body/mesh, S-Steel; body, 200 mm × 50 mm. For the analysis of the chemical nature of the collected particles Attenuated total reflection—Fourier transform infrared (ATR-FTIR) spectrometry (spectrometer: Alpha ATR unit, Bruker 27; equipped with a diamond crystal for measurements) was used. Spectra were taken from 4000 to 400 cm^−1^ (resolution 8 cm^−1^, 16 accumulated scans, Software OPUS 7.5) and compared with entries from an in-house database described previously^24^ or the database provided by the manufacturer of the instrument (Bruker Optik GmbH, Leipzig, Germany). This comparison of the IR-spectra allowed to distinguish biodegradable from conventional plastic fragments, but also from residues of other materials including unknowns. An incident light microscope (microscope, Nikon SMZ 754T; digital camera, DS-Fi2; camera control unit, DS-U3; software, NIS Elements D) was used for visual documentation of all particles identified by ATR-FTIR as synthetic plastics (biodegradable or otherwise).

### Analysis of plastic fragments in the liquid fertilizers

The liquid fertilizer samples were also sieved with 5 mm and 1 mm sieves to obtain possibly present fragments > 1 mm. For the preparation of the plastic fragments < 1 mm (down to 10 µm) an adjusted enzymatic-oxidative digestion method based on a method suggested by Löder et al. 2017 was adapted^25^. For this, the liquid fertilizer sample was mixed well with a metal rod and 50 mL were quickly poured into a 300 mL glass beaker (Schott-Duran). The metal rod and the glass beakers were washed in advance with Millipore water. Then 50 mL of a 10 wt% sodium dodecyl sulfate (SDS) solution (≥ 95 % SDS; Karl Roth) was added and the mixture incubated at 50 °C for 72 h under gentle agitation (Universal Shaker SM 30 B, Edmund Bühler GmbH, Bodelshausen, Germany). Subsequently, 2 × 25 mL of 30% hydrogen peroxide was slowly added under a fume hood. Since the reaction of hydrogen peroxide with organic matter is highly exothermic, an ice bath was used to keep the reaction temperature below 40 °C. Once the reaction had subsided and the mixture had again reached room temperature, the solution was filtered over a 10 µm stainless-steel-mesh filter (47 mm diameter, Rolf Körner GmbH, Niederzier, Germany) with a vacuum filtration unit (3-branch stainless-steel vacuum manifold with 500 mL funnels and lids, Sartorius AG, Göttingen, Germany). All filtrations were conducted under a laminar flow hood to minimize contamination with microplastics from the surrounding air. All matter retained by the filter was rinsed with filtered (0.2 µm) deionized water to remove residual chemicals. Afterwards, the retained matter was rinsed into a fresh 300 mL glass beaker with approximately 50 mL of 0.1 M Tris-HCl buffer (pH 9.0). As particles tended to adhere to the stainless-steel filter, the filter was also placed into the beaker. Ten milliliters of Protease A-01 solution were added and the beaker was incubated at 50 °C for 12 h with gentle agitation. Afterwards, the filter was thoroughly rinsed off into the beaker with filtered deionized water to recover any adhering particles and then used to filter the incubated solution. The retained matter was rinsed into a fresh glass beaker with 25 mL of 0.1 M NaAc buffer (pH 5). The filter was again placed in the jar as well, 5 mL of the Pektinase L-40 solution was added, and the beaker was incubated for 72 h at 50 °C. The filter was rinsed and used to filter the sample as before. Any matter retained by this filtration step was again rinsed into a fresh glass beaker with 25 mL of 0.1 M NaAc buffer (pH 5). The filter was again placed in the beaker, 1 mL of a Viscozyme L solution was added, and the jar was incubated at 50 °C for 48 h. The sample was filtered and the retained matter was transferred into 25 mL of a 0.1 M NaAc buffer (pH 5). Five mL of Cellulase TXL solution was added and the jar was incubated at 40 °C for 24 h.

Only after the enzymatic digestion were the preparations oxidized with Fenton’s reagent. This combination of enzymatic digestion and Fenton oxidation was necessary since for these types of samples Fenton treatment alone was not sufficient to remove enough of the organic material to allow µ-FTIR imaging. A detailed analysis of the challenge of sample preparation for µ-FTIR in case of complex samples has recently been published by some members of our group^33^, where further details can be found.

For this purpose, the mixture was filtered and the matter retained by the filter rinsed into a fresh glass beaker with ca. 20 mL filtered deionized water. Then, 20 mL of 30% H_2_O_2_ solution was added, and the mixture continuously stirred with a magnetic stir bar under the fume hood while adding 20 mL of 0.05 M Fe(II) solution (7.5 g of iron(II) sulfate heptahydrate (FeSO_4_ · 7 H_2_O) in 500 mL ultrapure water and 3 mL of concentrated sulfuric acid). An ice bath was again used to keep the reaction temperature below 40 °C. After approximately 2 h, the reaction had subsided, and the reagents were filtered off over a 10 µm stainless-steel-mesh filter. Residual Fenton’s reagent was removed by rinsing the filter retentate with filtered deionized water.

This treatment was followed by a density separation step with an aqueous zinc chloride solution. For this, the retained matter was transferred from the filter into a clean glass beaker using a metal spatula and approximately 50 mL ZnCl_2_ solution (ρ = 1.8 g cm^−3^) was added. The mixture was stirred with a magnetic stir bar until all aggregates were dispersed. Then, the mixture was transferred into a straight-walled separation funnel with a capacity of 400 mL. The mixture was stirred for several minutes with a glass rod and left to settle overnight (at least 12 h). Any plastic fragments present in the sample separate from any mineral matter by rising to the top. After release of the sediment, the low density particle fraction was filtered onto a new 10 µm stainless-steel-mesh filter, which was then rinsed with 98% filtered ethanol and filtered deionized water to remove residual ZnCl_2_.

Depending on the initial amount and the quality of its matrix, the number of particles recovered by the purification can vary. In order to avoid matrix interference, which would make FTIR analysis impossible, the aluminum oxide sample carrier filters (0.2 μm, Anodisc, Whatman GE Healthcare) must not be overloaded. Therefore, samples with a high amount of matter were suspended in filtered deionized water, evenly filtered over a 5 µm pore stainless steel-mesh filter (diameter: 47 mm), and then halved using custom made pliers that divide the circular filter in half. One half was washed into a clean 100 mL beaker, while the other was kept as backup sample. This process was repeated as often as necessary to achieve a subsample that could be transferred onto 3–5 aluminum oxide filters for spectroscopic measurement. The filters were analyzed with focal plane array-based µ-FTIR spectroscopy^24^, which allows the determination of the fragment shape, size, color and polymer type (again via the IR spectrum), using a Bruker Hyperion 3000 FTIR microscope (Bruker Optik GmbH) equipped with a 64 × 64 pixel FPA detector in conjunction with a Tensor 27 spectrometer. The samples were measured in transmission mode with a 3.8 × IR objective (spatial resolution 11.05 µm per pixel) and a wavelength range of 3600–1250 cm^−1^ (resolution 8 cm^−1^, 6 accumulated scans). Data processing was conducted using Bruker OPUS software version 7.5 (Bruker Optik GmbH) and automated spectral analysis was performed with the “BayreuthParticleFinder” module in ImageLab version 4.1 (EPINA GmbH, Güttersloh, Germany) based on Random Forest Decision Classifiers^34^ for 22 different polymer types.

### Analysis of the material properties of the various plastic materials

FTIR spectroscopy was used to directly compare the material properties of the BDP fragments, the commercial biodegradable bags, and the reference materials. The measurement was performed on either a Digilab Excalibur Series FTIR spectrometer (range 4000 to 550 cm^−1^, resolution ~4 cm^−1^, 16 accumulative scans) or a PerkinElmer Spectrum 100 FTIR spectrometer (range 4000 to 450 cm^−1^, resolution 4 cm^−1^, 4 accumulative scans).

The polymer content and composition of bags and fragments were quantified by ^1^H NMR in CDCl_3_ with 64 scans using a 300 MHz Bruker Ultrashield 300 spectrometer. MestreNova software was used for evaluation. 1,2-dichloroethane (DCE), which shows a single peak at 3.73 ppm, served as an internal standard. Proton peak integration of the areas at chemical shifts of 4.37–4.43 ppm (abbreviated as A_T_, methylene in BT units), 4.08–4.14 ppm (abbreviated as A_A_, methylene in BA units), 5.12 ppm (abbreviated A_L_, methine in lactide units), and 3.73 ppm (abbreviated as A_STD_, methylene in the internal standard DCE) were used to calculate the respective masses of PBAT and PLA in the residue according to:

$${\mathrm{m}}_{\mathrm{PBAT}} ={\mathrm{n}}_{\mathrm{BT}}*{\mathrm{M}}_{\mathrm{BT}} + {\mathrm{n}}_{\mathrm{BA}}*{\mathrm{M}}_{\mathrm{BA}}$$

$${\mathrm{m}}_{\mathrm{PBAT}} = \left({\mathrm{A}}_{\mathrm{T}}*{\mathrm{M}}_{\mathrm{BT}} + {\mathrm{A}}_{\mathrm{A}}*{\mathrm{M}}_{\mathrm{BA}}\right)*\frac{{\mathrm{m}}_{\mathrm{STD}}}{{\mathrm{M}}_{\mathrm{STD}}*{\mathrm{A}}_{\mathrm{STD}}}$$

$${\mathrm{m}}_{\mathrm{PLA}} = {\mathrm{n}}_{\mathrm{LA}}*{\mathrm{M}}_{\mathrm{LA}} = 4*{\mathrm{A}}_{\mathrm{L}}*{\mathrm{M}}_{\mathrm{LA}}*\frac{{\mathrm{m}}_{\mathrm{STD}}}{{\mathrm{M}}_{\mathrm{STD}}*{\mathrm{A}}_{\mathrm{STD}}}$$

where m_PBAT_ is the mass of PBAT; n_BT_ and n_BA_ correspond to the moles of the BT and BA units of PBAT, respectively; M_BT_ and M_BA_ are their molar masses; m_PLA_ is the mass of PLA; n_LA_ corresponds to the moles of the lactic acid unit; M_L_ is the corresponding molar mass; M_STD_ is the molar mass of the internal standard; and m_STD_ is the mass (amount) used in the measurement. In addition, the ratios of the BT and BA units within the PBAT fraction of a given sample were calculated from the ^1^H-NMR data.

DSC was performed using a DSC 204 F1 Phoenix from Netzsch Instruments from − 50 to 200 °C with a heating rate of 10 °C min^−1^ under nitrogen atmosphere with a flow rate of 20 mL min^−1^. Each measurement consisted of two full heating and cooling runs.

WAXS was performed on a Bruker D8 Advance diffractometer within 2*θ* ranges of 5°–60° (for reference PLA and PBAT) and 8°–45° (for BDP fragments from compost and the commercial bags) in transmission mode (step size = 0.05°, scanning rate 40 s step^−1^), and Cu Kα (λ = 1.54 Å) X-rays were used. Foils from the reference materials PBAT and PLA were prepared by heat pressing at 150 °C and 160 °C, respectively. The heat-pressed PLA was further annealed at 80 °C for 3 days to increase crystallinity.

### Extraction and quantification of residual plastic as bulk from compost samples

Residual PBAT and PLA matter corresponding to fragments < 1 mm were extracted in bulk from compost samples using a previously published method^35^ in modified form. Compost aliquots were first sieved through a 1 mm mesh to remove the larger fragments, and then dried at 60 °C for 48 h prior to extraction. One hundred grams of material was placed in 500 mL glass bottles and 250 mL of a 90/10 vol% chloroform/methanol mixture was added. The glass bottles were sealed, placed on a horizontal shaker for 10 min and subsequently sonicated in a water bath at room temperature for 10 min. Afterwards, the containers were placed overnight in a fume hood. The next day, the contents were passed through a Büchner funnel under vacuum, and the retained residues were washed with excess chloroform to remove any remaining dissolved material. The solvents were removed from the filtrate by rotary evaporation under vacuum and the obtained residue was dried overnight in an oven at 45 °C under vacuum. To quantify polymer content and composition, ^1^H-NMR spectra were recorded for each extract. 1,2-dichloroethane was chosen as inner standard since it has a single peak at d = 3.74 ppm and thus does not interfere with the peaks of PLA and PBAT (see Suppl Figure S4. The peaks assigned to PLA or PBAT in a spectrum were integrated. As the peak intensity of ^1^H-NMR is proportional to the number of protons in a molecule, the integration values of peaks can be used for quantification purpose. The amounts of PBAT and PLA calculated for the extracts were then correlated to the dry weight of the extracted compost sample and used for the calculation of the total mass concentration (wt%) of PBAT and PLA per unit of dried compost.

## Supplementary Information

Supplementary Information.

## Data Availability

All data are available in the main text or the supplementary materials.
