# Supplementary material for: Municipal biowaste treatment plants contribute to the contamination of the environment with residues of biodegradable plastics with putative higher persistence potential
Source: Sci Rep. 2022 May 30;12:9021. doi: 10.1038/s41598-022-12912-z (PMC9151778; doi:10.1038/s41598-022-12912-z)
Supplement: Supplementary file 1 — Supplementary Information. [file 41598_2022_12912_MOESM1_ESM.docx]

**Supplementary materials**

**Biodegradable plastics in municipal biowaste treatment plants: pathway into the environment for microplastics with putative higher persistence potential**

**Authors**

Thomas Steiner^1†^, Yuanhu Zhang^2†^, Julia N. Möller^3†^, Seema Agarwal^2^, Martin G.J. Löder^3^, Andreas Greiner^2‡^, Christian Laforsch^3‡^, Ruth Freitag^1‡^*

^1^Process Biotechnology, University of Bayreuth

^2^Macromolecular Chemistry II, University of Bayreuth

^3^Animal Ecology I & BayCEER, University of Bayreuth

**Affiliations**

† These authors contributed equally to the experimental part of the described work

‡ Shared senior authorship

* Corresponding author address: Process Biotechnology, University of Bayreuth, Universitätsstrasse 30, 95440 Bayreuth, Germany, phone: +49 921 557371, Fax: +49 921 557375, e-mail: ruth.freitag@uni-bayreuth.de

**Content**

5 supplementary figures

1 supplementary table

2 supplementary references

**Supplementary figures**


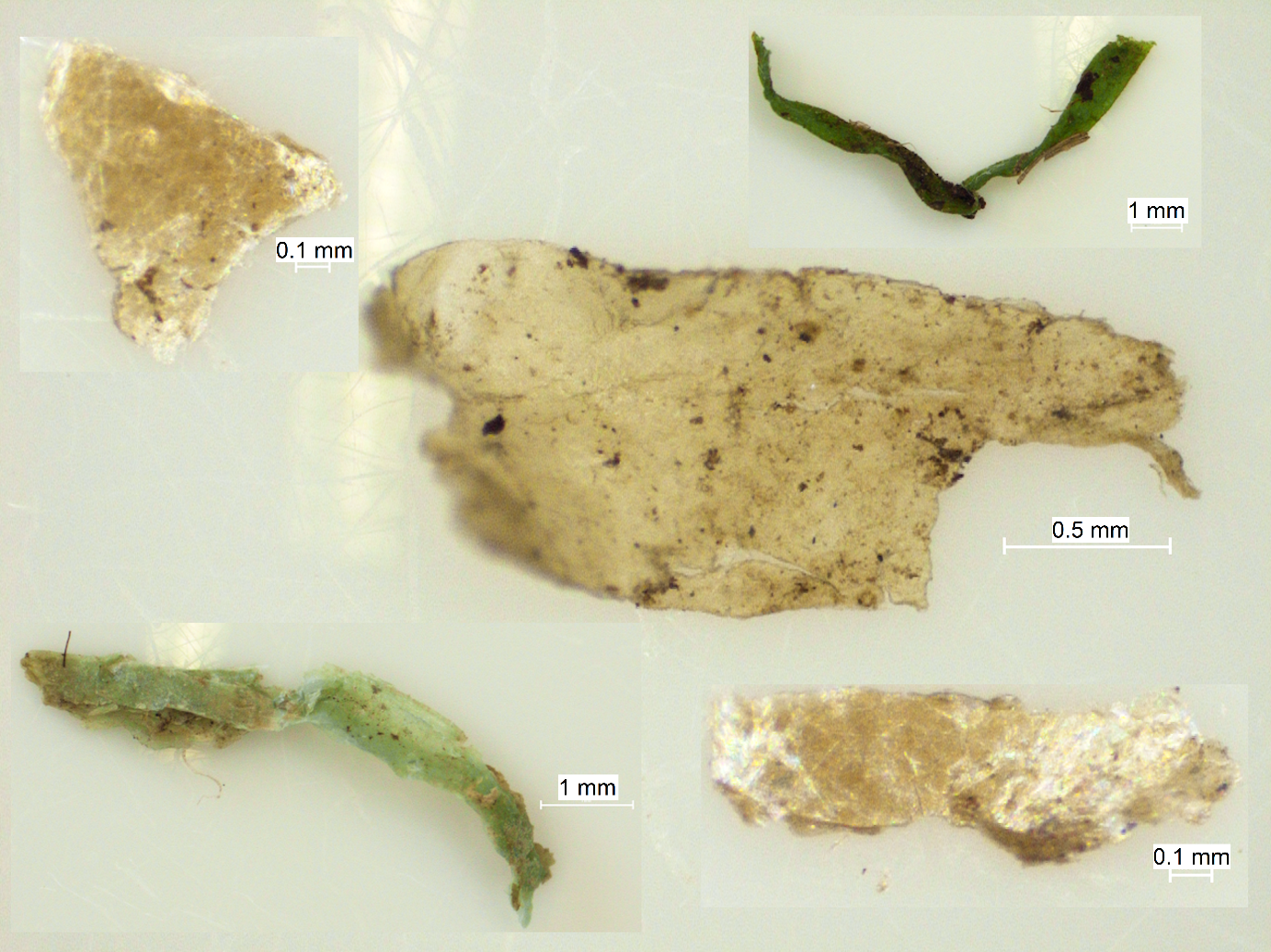


**Fig. S1.** Examples for plastic fragments found in composts.


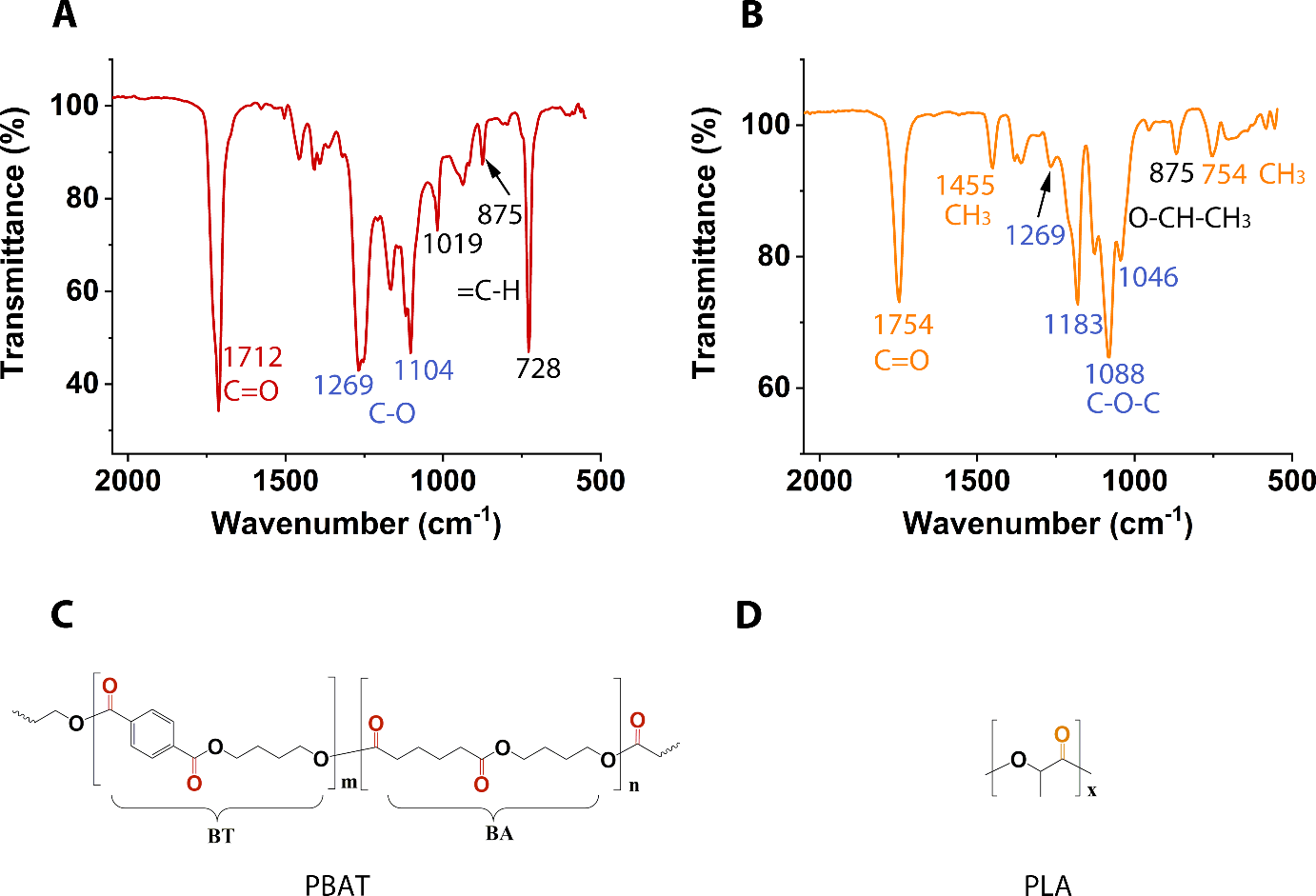


**Fig. S2.** FT-IR of references **(A)** PBAT and **(B)** PLA, and the chemical structures of **(C)** PBAT and **(D)** PLA. Representative functional groups of PBAT and PLA are indicated in their spectra in the same color. Peaks at 1754 cm^-1^ for the C=O group of PLA and at 1712 cm^-1^ for the C=O group of PBAT can easily differentiate these two polymers.

In these spectra, specific peaks at 1754, 1455, 1390, 1364, 1183, 1088, 1046, 952, 875, and 754 cm^-1^ were assigned to PLA. Among them, the peaks at 1754 cm^-1^ and 754 cm^-1^, representing C = O stretching and wagging of α-CH_3,_ respectively, can also be used to identify PLA in mixtures. The peaks at 1269, 1183, 1088, and 1046 cm^-1^ correspond to C－O－C stretching. The peak at 1455 cm^-1^ was assigned to the asymmetric bending of CH_3_, while the peaks at 1390 cm^-1^ and 1364 cm^-1^ correspond to the symmetric bending of CH_3_ and CH. The peak at 875 cm^-1^ is related to the absorption of O－CH－CH_3_. PBAT shows peaks at 2960, 2874, 1712, 1580, 1506, 1455, 1411, 1364, 1269, 1104, 1019, 875, and 728 cm^-1^, with the peaks at 2960 cm^-1^ and 2874 cm^-1^ corresponding to the stretching of CH_3_ and CH_2_, the peak at 1712 cm^-1^ to the C = O stretching, that at 1455 cm^-1^ to the in-plane bending of CH_2_, that at 1364 cm^-1^ to the out-of-plane bending of CH_2_, that at 1411 cm^-1^ to the O－CH_2_ bending, those at 1269 and 1104 cm^-1^ to the C－O stretching, those at 1580 and 1506 cm^-1^ to the skeleton vibration of benzene, that at 1019 cm^-1^ to the =C－H in-plane bending in the benzene ring, and those at 875 and 728 cm^-1^ to the =C－H-out-of-plane bending of benzene (*S1, S2*).

**
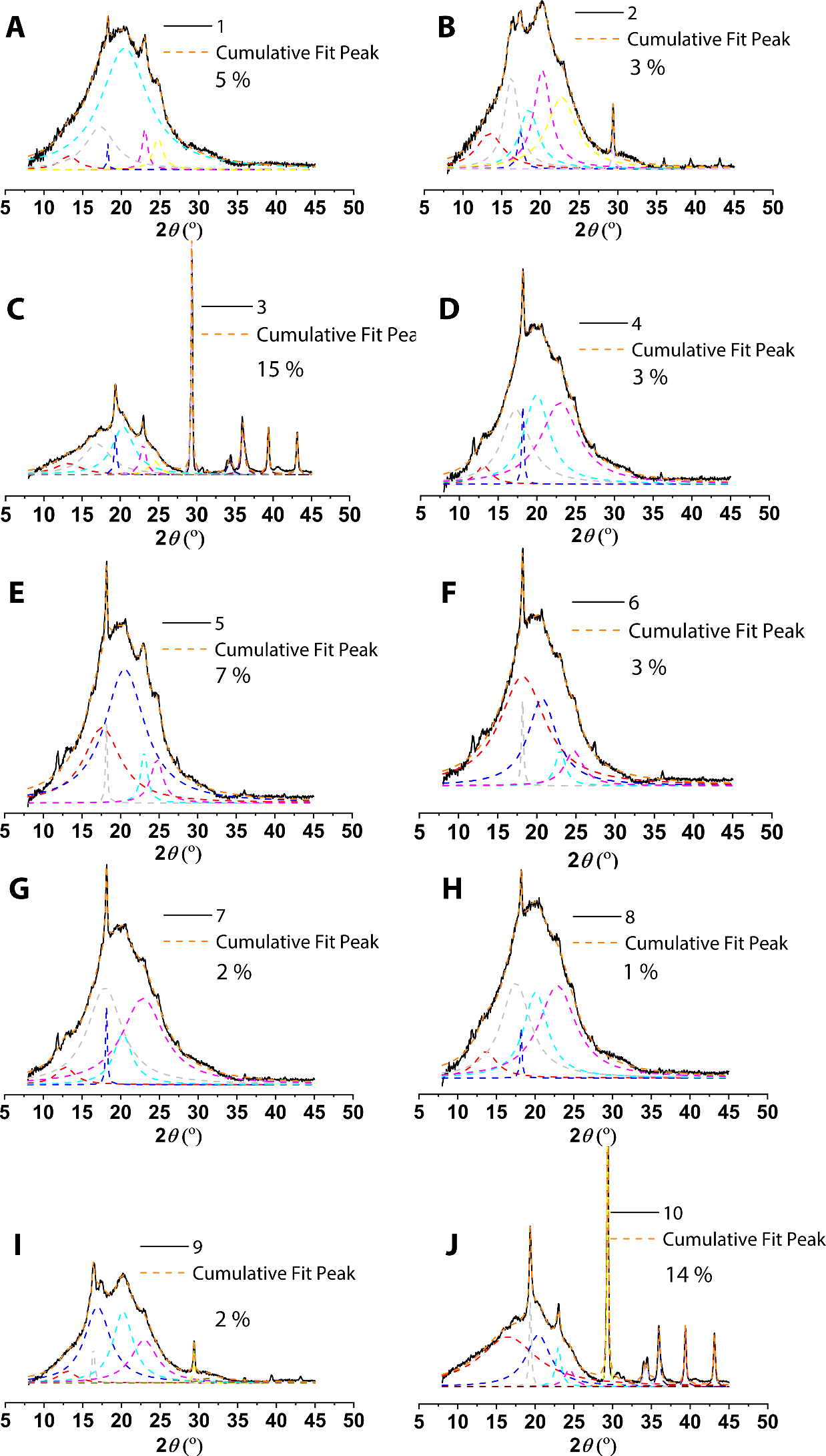
**

**Fig. S3.** WAXS curves and Lorenz fitting of commercial bags No. 1 **(A)** to No. 10 **(J)**; the percent value indicates the degree of crystallinity. The dash lines are the fitting peak curves for the XRD spectrum. Crystallinity can be obtained by dividing the integration area of the fitted peaks by the integration area of the entire spectrum.


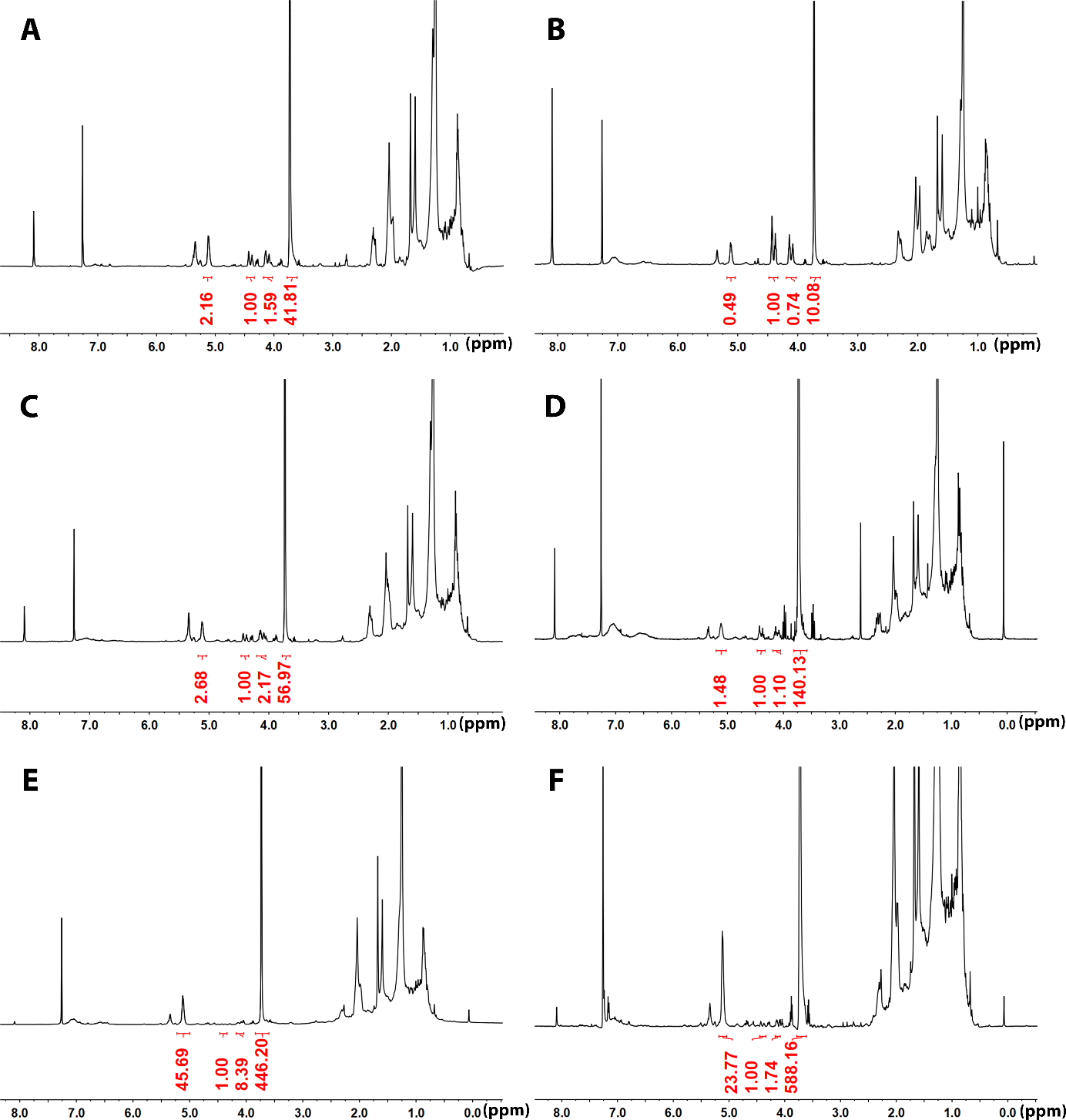


**Fig. S4.** ^1^H NMR of the extracted PLA and PBAT from f#1 **(A)**, f#2 **(B)**, p#3 **(C)**, f#3 **(D)**, p#4 **(E)**, and f#4 **(F)**; 1,2-dichloroethane was used as the internal standard (chemical shift, 3.73 ppm).


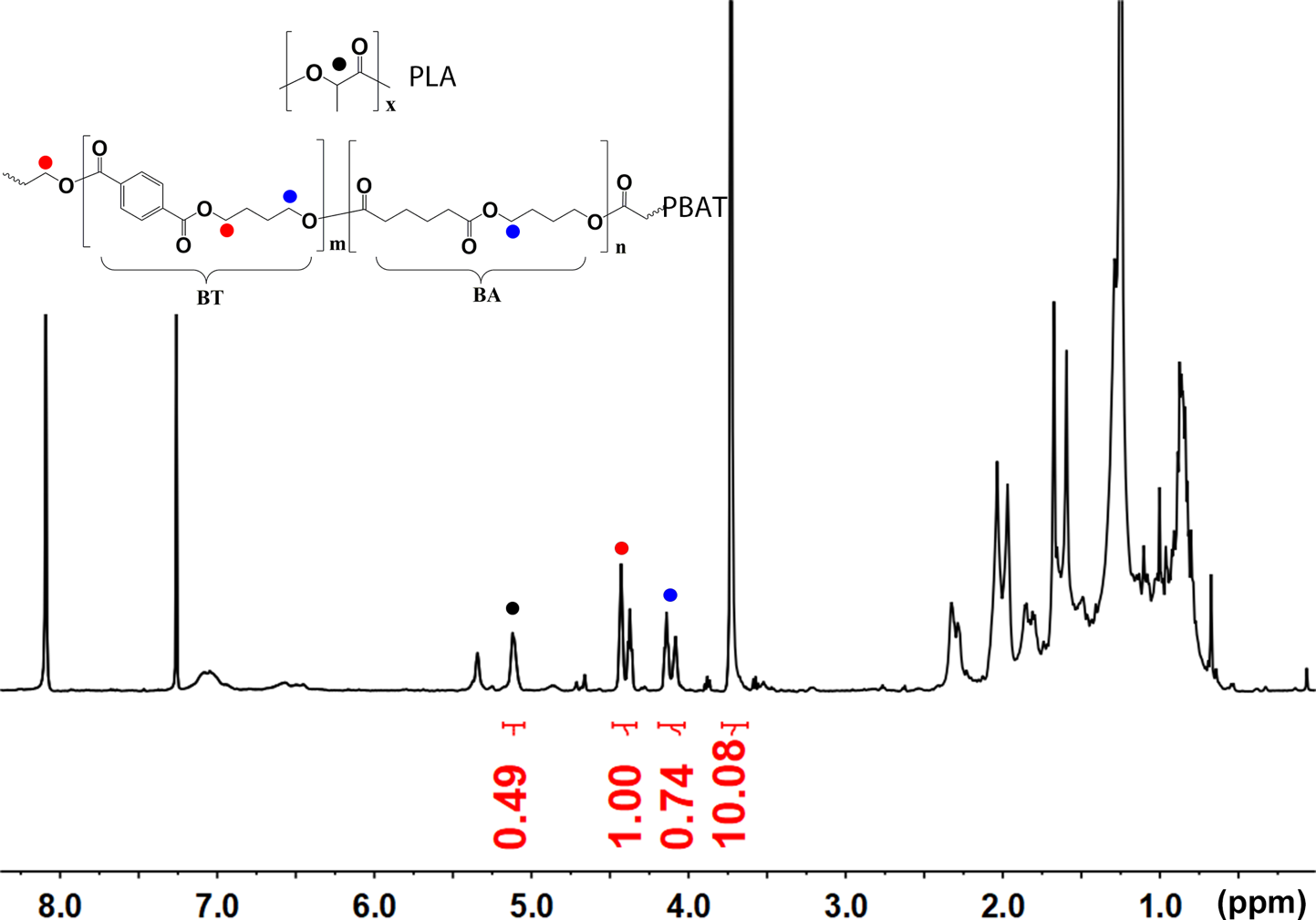


**Fig. S5.** Example of quantification of extracted PBAT and PLA from f#2 compost.

**Supplementary Tables**

**Table S1.** Bags certified as compostable, bought from local supermarkets for our investigation

| **Nr.** | **Supermarket chain** | **Brand of Bag** |
| --- | --- | --- |
| 1 | Real | Pely |
| 2 | Real | real Bio |
| 3 | ALDI | alio |
| 4 | Netto | Priva |
| 5 | Edeka | swirl |
| 6 | Edeka | Gut & Günstig |
| 7 | REWE | REWE |
| 8 | LIDL | purio |
| 9 | DM | Profissimo (dm) |
| 10 | Kaufland | Classic |

**Supplementary references**

S1 J. Coates, Interpretation of Infrared Spectra, A Practical Approach in *Meyers, R.A. (ed.) Encyclopedia of analytical chemistry,* pp. 10815 – 10837 (Wiley, Chichester, 2000).

S2 Y. Cai, J. Lv, J. Feng, Spectral Characterization of Four Kinds of Biodegradable Plastics: Poly (Lactic Acid), Poly (Butylenes Adipate-Co-Terephthalate), Poly (Hydroxybutyrate-Co-Hydroxyvalerate) and Poly (Butylenes Succinate) with FTIR and Raman Spectroscopy. J. Polym. Environ. 21, 108–114 (2013). doi: 10.1007/s10924-012-0534-2
